# Comparing Revision Total Knee Arthroplasty Stems at a High-Volume Revision Center

**DOI:** 10.3389/fsurg.2022.716510

**Published:** 2022-03-11

**Authors:** Bernard P. Kemker, Christopher B. Sowers, Raees Seedat, Jibanananda Satpathy, Nirav K. Patel, Daniel J. Lombardo, Gregory J. Golladay

**Affiliations:** ^1^Department of Orthopaedic Surgery, VCU Health, Richmond, VA, United States; ^2^Virginia Commonwealth University, School of Medicine, Richmond, VA, United States

**Keywords:** stem fixation, revision total knee arthroplasty (rTKA), cemented stem, hybrid stem, press-fit stem

## Abstract

**Introduction:**

Hybrid fixation and fully cemented fixation are commonly used in revision total knee arthroplasty (rTKA). These two techniques are typically done based on surgeon preference and one has not demonstrated superiority over the other. The purpose of this study was to examine if there was a difference in survivorship between the two different techniques.

**Methods:**

A retrospective cohort study of all consecutive patients undergoing rTKA (CPT 27487) from January 1, 2011 to January 1, 2018 at a single academic center was performed. Patients were divided into cemented and hybrid rTKA groups with comparison of patient demographic, clinical and radiological outcomes, reoperation, change in post-operative hemoglobin (HgB), and length of stay (LOS).

**Results:**

A total of 133 rTKA for 122 patients were identified: 30.1% in the cemented and 69.9% in the hybrid groups. There was no significant difference in age (*p* = 0.491), sex (*p* = 0.250), laterality (*p* = 0.421), or body mass index (BMI) (*p* = 0.609) between the two groups. Mean LOS (hybrid 4.13 days, cemented 3.65 days; *p* = 0.356) and change in Hgb (hybrid 2.95 mg/dL, cemented 2.62mg/dL; *p* = 0.181) were not statistically different between the groups. Mean follow up for the hybrid (25.4 months, range 2–114 months) and cemented (24.6 months, range 3–75.5 months) rTKA was not statistically significant (*p* = 0.825). Overall survival rates were 80.9% in the hybrid and 84.6% in the cemented groups (*p* = 0.642).

**Conclusions:**

Hybrid and fully cemented rTKA techniques have similar survival rates at a minimum followup of 2 years. Additionally, in our cohort, age, gender, and BMI were not associated with failure in either group. Furthermore, we did not observe differences in LOS or change in hemoglobin suggesting early postoperative complications may not differ between cemented and hybrid stemmed groups. Continued long-term research is required for defining the best rTKA technique.

## Introduction

Fixation techniques in revision total knee arthroplasty (rTKA) are still controversial. The main concerns in revision total knee arthroplasty (rTKA) arise from bone loss secondary to implant failure and subsequent removal. Revision stems have a history of success in addressing structural concerns when implant fixation is challenging (1–6). Traditionally, stems used for fixation during rTKA are either press-fit with cement in the metaphysis and under the implants in a hybrid technique or are fully cemented (7).

Cemented stems have been noted to have good initial stability with long-term fixation in stems as short as 30 mm (8–11). Additionally, cemented stems may accommodate canals of various deformities, especially in those with pronounced bone loss (12). It is also hypothesized that cemented stems may better seal off the canals and reduce postoperative blood loss. Some potential drawbacks include concern for stress shielding and an increase in bone loss if re-revision is needed (13, 14).

Alternatively, hybrid stems achieve primary fixation through their diaphyseal stem-bone fixation and secondarily with epiphyseal or metaphyseal cement. The stem position can dictate the position of the femoral and tibial components, thus potentially necessitating the use of offset stems. Press-fit stems used for hybrid fixation demonstrate improvements in pain and functional scores and long-term survival in some studies (6, 15–17). However, there are other studies reporting higher tibial stem tip pain, leading to lower knee scores and decreased patient satisfaction. Additionally, press-fit stems are associated with increased rates of periprosthetic fractures (18–21).

Despite long-term use in rTKA, high quality evidence comparing cemented stems to hybrid stems is scarce, making it difficult to differentiate between the two (1, 7, 22, 23). The consensus is that no significant differences exist between cemented and hybrid stems. Therefore, the primary objective of this study is to compare cemented and hybrid stems in rTKA, looking primarily at survivorship at final follow-up. The secondary objective will be to compare demographic factors that may be related to failure, in addition to blood loss and length of stay (LOS).

## Materials and Methods

This was a retrospective review of prospectively collected institutional data at an academic center with a large revision arthroplasty case load. All consecutive patients undergoing rTKA of both components (CPT 27487) between January 1, 2011 and January 1, 2018 were included. These included patients with cones, sleeves, unlinked constrained, and linked constrained components. Patients were excluded if they did not have both femoral and tibial components revised, did not have both femoral and tibial stem fixation, or the patient was lost to follow-up before 2 years. Medical records and radiographs were evaluated for all patients, and demographics including age, sex, body mass index (BMI), and laterality were recorded for each group.

**Figure 1:**
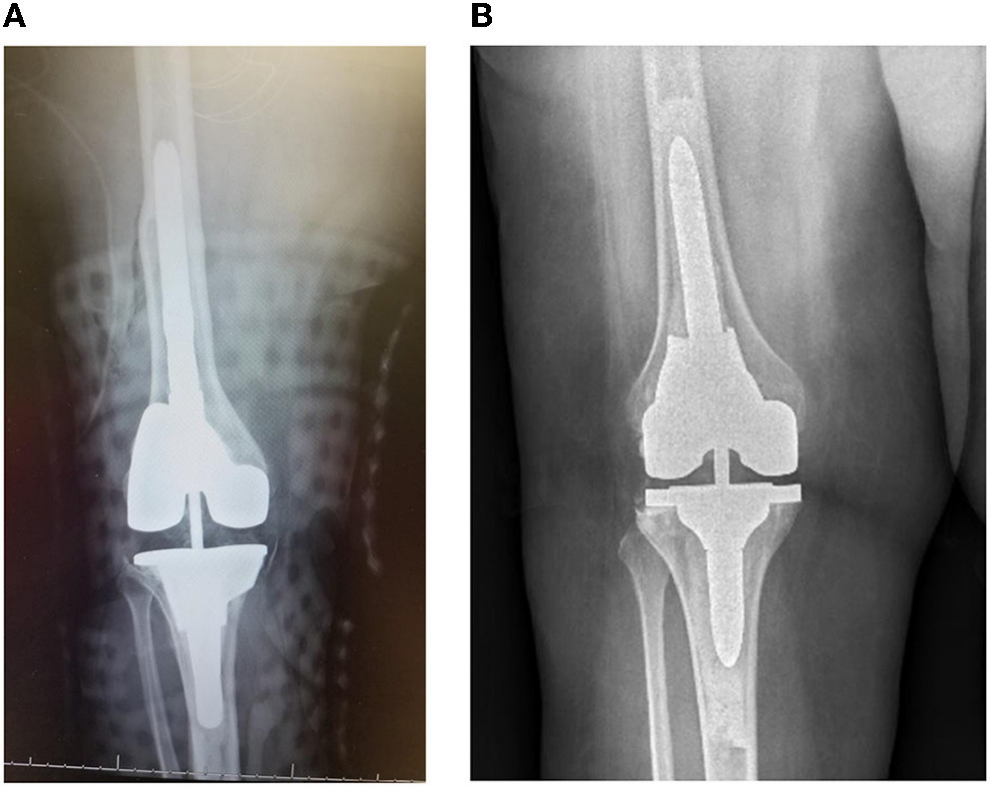
**(A)** Depicts a hybrid RTKA fixation technique and **(B)** depicts the fully cemented RTKA technique. The hybrid technique has cement only in the metaphysis with cortical engaging stems within the diaphysis. In comparison, the fully cemented technique has cement fixation in both the diaphysis and the metaphysis.

The decision for fully cemented vs. hybrid fixation was determined by the senior surgeon. All the implants were pre-coated with cement prepared with a 4th generation technique to unitize the construct. After appropriate reaming, a cement plug for a 2-cm distal cement mantle was placed for the cemented fixation. The canals were then filled retrograde with cement and pressurized. For the hybrid fixation, diaphyseal reaming continued until the reamer outer diameter matched the inner diameter of the diaphysis. The diaphyseal stem was placed in a “line-to-line” method based on the size of the largest used reamer. Vacuum mixed cement was placed around the metaphyseal portions of the implants and pressurized into the metaphyseal bone, taking care to leave the canal open and free of cement. The hybrid implant was then inserted taking care to pressurize all epiphyseal and metaphyseal cement during implant impaction. Augments and constraints were used on a case-by-case basis when clinically indicated. In both fully cemented and hybrid fixation, the main goals of rTKA were joint line restoration, alignment according to the mechanical axis, a balanced knee, and a well tracking patella. Hybrid fixation and cemented fixation techniques were illustrated in Figure 1.

The primary objective was to assess the implant survivorship of cemented vs. hybrid stems. Failure was defined as any surgical re-operation on the rTKA, such as explant, amputation, polyethylene exchange, lysis of adhesions, fracture fixation, extensor mechanism reconstruction, and repeat rTKA. Time to failure between the fully cemented and hybrid rTKAs were compared via Kaplan-Meier plots. BMI, age, gender, and laterality were evaluated for possible correlations with early failures between the two techniques. Secondary endpoints of LOS and change in hemoglobin (HgB) (preoperative subtracted by post-operative nadir) were compared between the hybrid and fully cemented rTKAs. The secondary objective was to compare change in Hgb and LOS between the two groups for possible correlations with early failures. Patients underwent routine postoperative clinical follow-up.

Statistical analysis was performed using Microsoft Excel (Microsoft Corporation; Redmond, WA). Student *t*-tests were utilized to compare continuous variables between the fully cemented and hybrid rTKAs. Categorical variables were compared between groups with a chi-squared test. A logrank test was used to examine whether survival of the rTKAs was statistically different between the groups. Patient BMI, age, gender, and laterality were compared with student *t*-tests and chi-squared tests between the fully cemented and hybrid fixation groups. Additionally, BMI, age gender, and laterality were compared within the fully cemented and hybrid fixation groups to identify any statistical correlation for failure with student *t*-tests and chi-squared tests. The secondary endpoints (LOS and change in HgB) were also analyzed between the fully cemented and hybrid fixation groups with student *t*-tests to identify any statistical correlation for failure. Statistical significance was set at *p* < 0.05.

## Results

**Figure 2:**
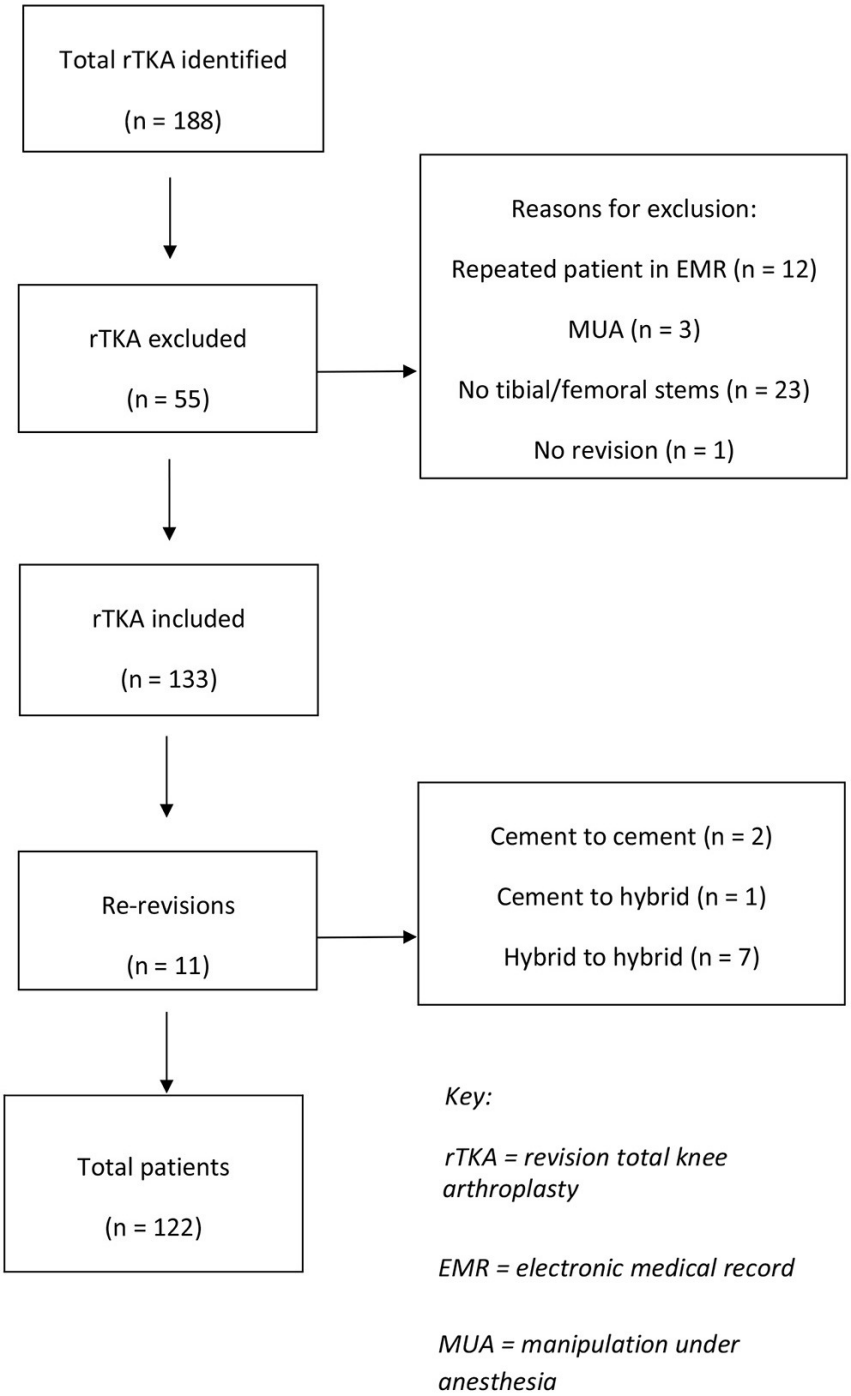
rTKA flow diagram.

A total of 188 rTKA under CPT were identified. Fifty-five knees were excluded, most commonly for being revisions without both femoral and tibial stems (*n* = 23), duplicate patients (*n* = 12), and falling outside the study time period (*n* = 10) (Figure 2). Of the 133 rTKAs, there were 40 fully cemented rTKAs (30.1%) and 93 hybrid rTKAs (69.9%).

**Figure 3:**
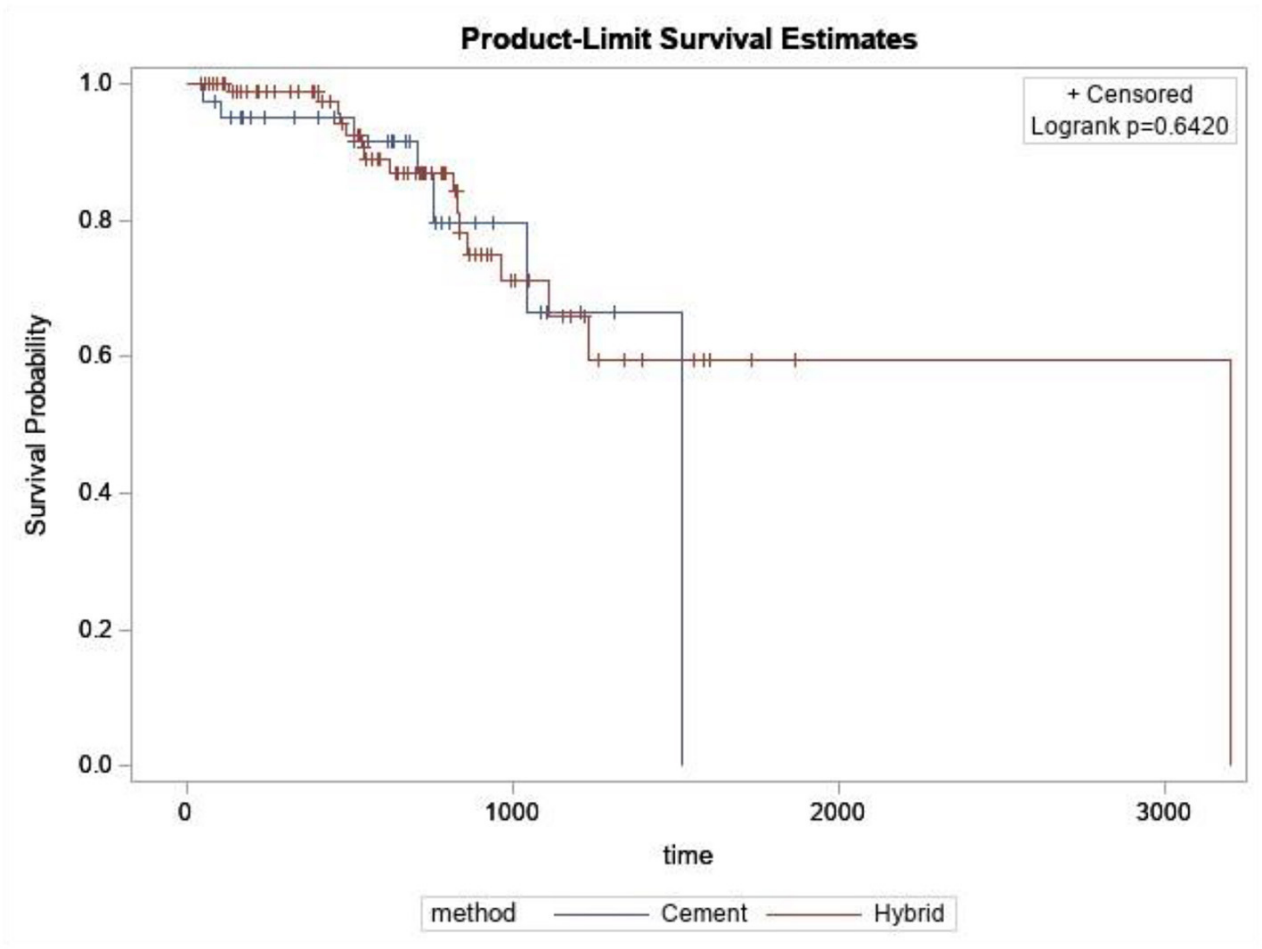
Kaplan Meier survival curves after log-rank analysis.

The patient demographics of the 133 patients can be found in Tables 1, 2. The overall survival rate was 81.8%.; Survival was 82.5% for the cemented cohort and 80.6% for the hybrid cohort which was not statistically significant (*p* = 0.642) (Figure 3). Descriptive information for the failed rTKAs is listed in Table 3. Age, sex, laterality, and BMI had no association with hybrid or cemented rTKA failure (Table 2). In both the fully cemented and hybrid cohorts, failed rTKAs had a significantly longer follow up than the surviving rTKAs (fully cemented *p* = 0.014; hybrid *p* < 0.005).

**Table 1:** Mean Patient demographics for fully cemented and hybrid cohorts.

	**Overall**	**Cemented**	**Hybrid**	
Age (years)	63.8 (range = 41–87)	63.8 (range = 45–85)	63.8 (range = 41–87)	*p* = 0.491
Sex (male)	47	13	34	*p* = 0.653
Laterality (right)	64	24	40	*p* = 0.018
BMI (kg/m^2^)	33.4 (range = 20.9–62.2)	32.7 (range = 21.6–49.6)	33.8 (range = 20.9–62.2)	*p* = 0.383
Follow up (months)	25.8 (range = 2–114)	24.6 (range = 3–75.5)	25.4 (range = 2–114)	*p* = 0.825

*BMI, body mass index*.

**Table 2:** Mean patient demographics for survival vs failure in cemented and hybrid cohorts.

	**Cement survival**	**Cement failure**		**Hybrid survival**	**Hybrid failure**	
Age (years)	64.2	61.7	*p* = 0.491	64.5	60.59	*p* = 0.144
Sex (male)	11	1	*p* = 0.250	28	7	*p* = 0.819
Laterality (right)	20	4	*p* = 0.421	34	6	*p* = 0.351
BMI (kg/m^2^)	32.3	33.8	*p* = 0.609	33.7	33.5	*p* = 0.918
Follow up (months)	21.8	38.0	*p* = 0.014	21.3	42.0	***p*** **= 0.005**

*BMI, body mass index. The bold values means statistically significant difference in follow up between hybrid stem survival and hybrid stem failure*.

**Table 3:** Description of Failed Hybrid rTKAs.

**Patient**	**Indication for revision**	**Cause of revision failure**	**Age**	**Sex**	**Side**	**BMI (kg/m^2)^**	**LOS (day)**	**HgB (g/dL)**	**Follow up (months)**
1	Aseptic loosening	Infection	74	F	R	36.0	4	4.8	8.8
2	Infection	Fracture/catastrophic failure at stem junction at osteotomy site	49	M	L	29.8	2	0.9	37.5
3	Malalignment	Loosening/metallosis	62	F	R	30.0	2	3.9	53.5
4	Instability	Instability	63	F	L	26.2	1	1.6	57.5
5	Aseptic loosening	Aseptic loosening	59	F	L	38.2	4	1.9	114.0
6	Infection	Infection	55	F	R	45.1	3	1.7	58.0
7	Midflexion instability	Arthrofibrosis requiring poly exchange at outside hospital	72	F	L	36.9	4	0.7	24.5
8	Malrotation	Malrotation/patella maltracking	47	M	L	41.7	4	4.1	40.5
9	Instability	Infection	60	M	L	36.0	2	3.9	16.3
10	Femoral fracture	Infection	60	F	L	26.3	2	1.2	28.0
11	Infection	Infection	63	M	R	30.7	4	4.8	15.0
12	Infection	Infection	74	F	L	25.8	6	3.6	24.5
13	Infection w/extensor mechanism repair	Infection	75	M	R	33.5	5	3.7	23.5
14	Aseptic loosening of tibial component	Infection	69	F	L	32.9	5	n/a	85.5
15	Pain	Instability	41	F	L	32.3	3	3.3	52.0
16	Infection	Infection	48	M	R	42.2	4	0.3	27.3
17	Infection	Infection	59	M	L	26.4	6	4.9	47.8

*BMI, body mass index (kg/m)^2^; LOS, length of stay (days); HgB, hemoglobin (g/dL); F, female; M, male; R, right; L, left*.

The overall mean LOS was 4.0 days (range: 0–15 days), with the mean LOS for the cemented and hybrid cohorts being 3.7 days (range: 0–15 days) and 4.1 days (range: 1–13 days) respectively (*p* = 0.352). The overall mean change in Hgb was 2.85 g/dL (range: 0–6.7 g/dL), with that for the cemented and hybrid cohorts being 2.62 g/dL (range: 0–4.7 g/dL) and 2.95 g/dL (range: 0–6.7 g/dL) respectively. Additionally, in the cemented cohort, the mean LOS was 3.1 days (range: 0–15 days) for stems which survived, compared to 5.4 days (range: 2–13 days) for those which failed (*p* = 0.087). In the hybrid cohort, the mean LOS was 4.3 days (range: 1–13 days) for stems which survived, compared to 3.6 days (range: 1–6 days) for those which failed (*p* = 0.301). In the cemented cohort, the mean change in HgB was 2.69 g/dL (range: 0.4–4.7 g/dL) for stems which survived, compared to 2.24 g/dL (range: 0.7–4.6 g/dL) for those which failed (*p* = 0.373). In the hybrid cohort, the mean change in HgB was 2.98 g/dL (range: 0.5–6.7 g/dL) for stems which survived, compared to 2.83 g/dL (range: 0.3–4.9 g/dL) for those which failed (*p* = 0.700).

## Discussion

Choosing between cemented and hybrid stem fixation for revision TKA is a complex decision. One must consider patient bone quality, surgeon preference, and theoretical advantages of a particular fixation technique when choosing between the two techniques. (7, 22, 23) Current research suggests there are no significant differences in outcomes between the two fixation techniques (1, 23). The results of this study also identified no significant difference in implant survival at 2 year follow-up. Additionally, there was no significant difference in LOS or change in HgB between groups. These findings further support comparable surgical outcomes between cemented and hybrid stems.

This study did not find any difference in survivorship between cemented and hybrid stems at short-term follow up, which is consistent with previously reported data. In 2017, Fleischman et al. analyzed 223 patients and found both techniques had a similar risk of mechanical failure when corrected for covariates (23). Further, a meta-analysis identified neither stem fixation technique to be superior in rates of failure, reoperation, infection, or aseptic loosening (1). Additionally, this study looked at changes in HgB and LOS as two possible perioperative indicators of early failure. We did not identify differences between the cemented and hybrid cohorts when analyzing the changes in HgB following surgery. While literature on changes in HgB in rTKA is scarce, a prior systematic review on blood loss in revision arthroplasty did not recognize stem fixation technique as a significant contributor to differences in blood loss (24). The mean LOS of 4 days was congruent with a prior study which reviewed 10,604 rTKA cases documented in the NSQIP database. This study identified increasing operative time as the main indicator of increased LOS (25). Further, our study did not find any patient characteristics that impacted implant survival.

The overall failure rate in this study was 18%, which was higher than reported in the literature. However, considering this study included both septic and aseptic revisions, and took place at a tertiary referral center with a complex patient population, we feel this failure rate to be comparable to similar studies that reported failure rates of 33–10% (23, 26, 27). While the bulk of the current literature has determined the two fixation techniques to be similar, there are studies that found the risk of radiographic loosening to be significantly less in a hybrid stem technique (26). However, this study also recognized the difficulty in eliminating selection bias, as it is likely the cemented cohort presented with worse bone quality, and thus, proved a more challenging operation with a lower chance of success.

There are several limitations to the study. Firstly, the study was a retrospective study done at a single institution with short-term follow-up. The design and non-randomized nature may have introduced some selection bias for the two groups. As a tertiary referral center, many of our patients travel from afar and do not follow up past 2 years unless there is an adverse outcome. Thus, we noticed decreasing compliance for continued follow up. Finally, while 133 rTKAs for inclusion is likely underpowered, this number is comparable to those reported in previous literature (26, 28). Subgroup analysis would have strengthened the paper, however, subgroup analysis with the number in our data set would have been small and unlikely to yield a clinically significant, even if statistically significant, result. While multiple implant companies were used in the study, comparison of all types of revision companies and the differences in component mechanisms such as stem size/diameter, polyethylene locking mechanisms, fixed bearing polyethylene, rotating platform, and mechanisms of linked and unlinked constraints, cones, and metaphyseal sleeves would have resulted in small cohorts where a clinically significant comparison would be underpowered. Continued research would be insightful in rTKA. Varus Valgus angle were not recorded as the amount of varus/valgus is depenendent on long leg films from hip to ankle, which was not present in most patients.

Our study has multiple strengths, notably the inclusion of all rTKAs. Many other studies exclude rTKAs that use cones, sleeves, or linked hinge devices. Thus, we believe that our data is more applicable clinically, as the application of metaphyseal fixation or linked constrained can change intraoperatively. Additionally, both septic and aseptic rTKA was included for analysis, while other studies exclude septic revisions (22, 27). Finally, we included multiply revised knees for analysis. Previous studies only analyzed primary to rTKA in terms of failure (22, 28); thus, our results are generalizable to the majority of orthopedic surgeons.

## Conclusions

This study demonstrates that hybrid and fully cemented rTKA have similar overall survival rates in the short-term. When comparing these techniques, there was no association between patient characteristics and implant survival or failure. However, larger, prospective, ideally randomized studies are required to corroborate these findings. Practically, these studies should include rTKAs for any indication, including those with prior rTKA.

## Data Availability Statement

The raw data supporting the conclusions of this article will be made available by the authors, without undue reservation.

## Ethics Statement

Ethical review and approval was not required for the study on human participants in accordance with the local legislation and institutional requirements. Written informed consent for participation was not required for this study in accordance with the national legislation and the institutional requirements.

## Author Contributions

All authors were involved in the conception and design of the study, data collection and analysis and interpretation, and preparation of manuscript.

## Conflict of Interest

The authors declare that the research was conducted in the absence of any commercial or financial relationships that could be construed as a potential conflict of interest.

## Publisher's Note

All claims expressed in this article are solely those of the authors and do not necessarily represent those of their affiliated organizations, or those of the publisher, the editors and the reviewers. Any product that may be evaluated in this article, or claim that may be made by its manufacturer, is not guaranteed or endorsed by the publisher.
